# Feeling signs: motor encoding enhances sign language learning in hearing adults

**DOI:** 10.1017/s0272263124000196

**Published:** 2024-04-29

**Authors:** Laura M. Morett, Mathew Cieśla, Mary E. Bray, Karen Emmorey

**Affiliations:** 1 University of Missouri;; 2 Northumbria University;; 3 University of Alabama;; 4 San Diego State University

## Abstract

Manual production enhances learning and recall of signs by hearing second language learners; however, the mechanisms enabling this effect are unclear. We examined whether the motor encoding (somatosensory feedback) that occurs during sign production benefits learning and whether it interacts with sign iconicity, which also enhances learning. American Sign Language (ASL) signs varying in iconicity were learned either via production (repetition) with the eyes closed or via observation without production. Signs learned via production were recalled more accurately than signs learned via observation, indicating that motor encoding from manual production enriches the representations of signs. Moreover, the effect of motor encoding interacted with iconicity, suggesting that motor encoding may particularly enhance the recall of signs low in iconicity. Together, these results reveal the importance of somatosensory feedback as a key mechanism underlying the beneficial effect of production on sign learning, demonstrating that feeling one’s own signing promotes learning and recall of signs.

## Introduction

When hearing individuals learn a sign language, they acquire a second language (L2) in a second modality (M2) (Chen Pichler & Koulidobrova, 2016). Despite growing interest, M2L2 acquisition is understudied relative to spoken L2 acquisition (Schönström, 2021). In addition to broadening the diversity of languages studied within the field of L2 acquisition, research on M2L2 acquisition can provide unique insight into the role of motor encoding in L2 learning and representation. Several studies suggest that deaf signers rely primarily on motor (somatosensory) rather than visual input from their own signing as a means of self-monitoring and error detection (Emmorey, Bosworth, et al., 2009). Moreover, producing actions and signs similarly improves memory for action phrases and nouns in hearing non-signers and deaf signers (Zimmer & Engelkamp, 2003). In addition, there is evidence that relative to observing hand gestures conveying the meanings of words from an unfamiliar spoken L2, producing such hand gestures enhances the learning of these words, suggesting that meaningful motor encoding during learning enriches L2 lexical representations (Garcia-Gamez & Macizo, 2019; Morett, 2018). These findings lead us to hypothesize that motor encoding may play an important role in sign learning during M2L2 acquisition. The current study tests this hypothesis by comparing sign learning via production without visual encoding vs. observation without production, providing insight into how somatosensory feedback enriches representations of M2L2 signs.

Embodied theories of language processing posit that the body plays an integral role in language comprehension, production, and acquisition (e.g., Atkinson, 2010). Although much of this research has focused on how conceptual representations are grounded in embodied experience (e.g., Dijkstra & Post, 2015), some studies have examined how the body’s involvement in language production affects language comprehension. For example, some evidence suggests that speech is comprehended by mentally simulating articulation (D’Ausilio et al., 2009; Iacoboni, 2008; but see Hickok et al., 2011) and that writing words by hand facilitates their subsequent recognition (Longcamp et al., 2008). With respect to sign language, evidence suggests that similar to speech, comprehension may entail mentally simulating articulation (Corina & Gutierrez, 2016; Watkins & Thompson, 2017; but see Brozdowski & Emmorey, 2020) and that producing signs during M2L2 learning enhances their subsequent recall (Morett, 2015). Taken together, these findings provide evidence that language comprehension is influenced by motor encoding from language production.

A key mechanism of embodied language processing is iconicity, which facilitates the association of symbols with their referents via form-meaning resemblance (Perniss & Vigliocco, 2014). Traditionally, the form of language and its underlying representations have been considered largely amodal and abstract, and iconicity has been considered marginal and largely irrelevant to language processing and acquisition (e.g., Newmeyer, 1992). Although this view was originally derived from spoken languages, it has been extended to sign languages to affirm their status as languages rather than gestural systems (Klima & Bellugi, 1979). Contemporary research with a more nuanced and expansive view of iconicity has refuted the traditional view by providing evidence of iconicity’s pervasiveness in both spoken and signed languages from around the world (Dingemanse et al., 2015; Perniss et al., 2010). Consistent with this view, iconicity facilitates the acquisition of L1 signs by deaf children from a variety of languages, including American Sign Language (ASL; Caselli & Pyers, 2017), British Sign Language (BSL; Thompson et al., 2012), Turkish Sign Language (Sümer et al., 2017), Israeli Sign Language (Novogrodsky & Meir, 2020), and the acquisition of L2 signs from German Sign Language by hearing children (Goppelt-Kunkel et al., 2023).

For adults, iconic gestures conveying the meanings of words from an unfamiliar L2 facilitate learning (Kelly et al., 2009; Morett, 2014). Moreover, iconic gestures may influence the degree to which M2L2 signs are perceived as iconic, for better or worse. On the one hand, sign-gesture resemblance may facilitate sign-referent association, as evidenced by increased guessability and recall of the meanings of signs that closely resemble gestures used to depict the same concepts. For example, the sign “to cut with scissors” in the Sign Language of the Netherlands resembles a pantomimic gesture (Karadöller et al., in press; Ortega et al., 2019). On the other hand, sign-gesture resemblance may interfere with the phonological representation of signs, as evidenced by the less accurate imitation of iconic signs resembling gestures such as BSL *to brush*, which resembles (but is not identical to) pantomiming brushing, compared with non-iconic signs with no resemblance to gestures, such as BSL *sister*, which is articulated by tapping a curled index finger extended from a fist to the nose twice (Ortega & Morgan, 2015a, 2015b). Thus, iconicity has been proposed to facilitate conceptual-semantic associations in M2L2 learning but interferes with phonological representations (Ortega, 2017). Although iconicity is related to transparency—the ability to infer the meanings of signs based on their form—signs may be considered iconic but may not be transparent. For example, in ASL, *ball* is articulated by bringing the hands together, depicting the shape of a ball, and is rated as highly iconic, but its meaning is not transparent (easily guessed) for non-signers (Sehyr & Emmorey, 2019). Thus, inferring the meanings of signs from gestures may not always benefit M2L2 learners. Experience with gestures may be transferred to representations of the phonological forms of similar signs in M2L2 acquisition, just as L1 speech sounds and syntactic structures are transferred to similar counterparts in spoken L2 acquisition (Morett & MacWhinney, 2013; Yang et al., 2022). However, gesture-sign transfer may be somewhat limited in early-stage M2L2 learners. For example, a pilot study eliciting signs from hearing non-signers revealed that handshapes used in gestures (e.g., a raised fist with index finger extended to express *wait*) were sometimes, but not always, transferred to signs with a similar handshape (Chen Pichler, 2011). Finally, the results of a recent experimental study suggest that although L2 sign iconicity positively affects meaning inference by hearing adult non-signers after 1–2 exposures, similarity to iconic gestures does not (Hofweber et al., in press).

In a previous study (Morett, 2015), we investigated the impacts of embodied action and iconicity on M2L2 sign learning by showing videos of signs from ASL that were high or low in iconicity and then prompting the production of either the signs or meaningless hand movements. We found that the production of signs during learning led to more accurate subsequent recall of learned signs than the production of meaningless hand movements. This finding is consistent with the encoding specificity principle (Tulving & Thompson, 1973), which posits that, relative to encoding contexts mismatching retrieval contexts, encoding contexts matching retrieval contexts facilitate recall. Furthermore, iconic signs were recalled more accurately than non-iconic signs, particularly following longer delays; however, this effect was not influenced by the meaningfulness of the embodied action (production of signs vs. meaningless hand movements). Although these findings suggest that motor encoding from sign production may facilitate M2L2 learning, visual encoding also occurred during sign production and may have affected learning. Moreover, although there was no evidence of an interaction between learning condition and iconicity, categorical variation in iconicity and a limited sample size (*n* = 26) may have precluded its detection. If such an interaction exists, the effects of iconicity and motor encoding may be (super-) additive, with motor encoding amplifying the effect of iconicity on M2L2 sign learning. Another possibility is that the effect of motor encoding may be compensatory, such that it is larger for more difficult-to-learn signs low in iconicity than for easier-to-learn signs high in iconicity. Alternatively, the effects of motor encoding and iconicity may not interact, indicating their independence. Thus, additional investigation is needed to understand how motor encoding affects M2L2 sign learning and whether—and how—it interacts with iconicity within this context.

In the current study, we manipulated motor encoding during learning by presenting ASL signs that varied continuously in iconicity (using ratings from the ASL-LEX database; Caselli et al., 2017; Sehyr et al., 2021), and then either prompting the repetition of these signs with the eyes closed (production condition) or presenting the signs for an additional time without production (observation condition). In the latter condition, we presented videos of the signs produced by the model rather than asking participants to imagine themselves signing as there was no way to verify whether participants complied with the instructions or whether the imagined signs were correct. Because our goal was to disentangle the impact of motor encoding from that of visual encoding, we did not include a condition incorporating both motor and visual encoding in the current study. We probed the recall of learned signs five minutes and one week after learning by asking learners to produce the signs given their English translation. On the basis of our previous finding that production during learning facilitates subsequent recall of learned ASL signs (Morett, 2015), we predicted that, relative to the observation condition, motor encoding without visual encoding would enhance subsequent recall of learned signs. Moreover, on the basis of previous work (e.g., Campbell et al., 1992), we predicted THAT iconicity would enhance sign recall, such that more iconic signs would be recalled more accurately than less iconic signs. Finally, we predicted that motor encoding would interact with iconicity, with the results distinguishing between a larger effect of motor encoding on the recall of more vs. less iconic signs.

## Method

### Participants

Fifty-two participants enrolled in a university in the southeastern US volunteered to participate in this experiment in return for partial course credit or pro bono. Nine were excluded due to technical issues (e.g., experimental control script crashing), one was excluded due to failure to follow instructions, and one was excluded due to correctly responding to less than half of the catch trials, yielding a final sample of 41 participants (4 males, 37 females; age: *M* = 19.28, SD = 1.32). This sample size exceeds by three the minimum sample size of 38 participants computed via a between-factors repeated measures power analysis based on Morett (2015) with 85% power to detect *f* =. 436 with α =. 05. All participants were L1 English speakers, with three reporting knowledge of a spoken second language. Thirty-three participants reported no knowledge of a sign language and eight reported minimal exposure to or knowledge of ASL (e.g., a few signs or the fingerspelled alphabet).^1^ This experiment was approved by the institutional review board of the university at which data were collected, and informed consent was obtained from all participants.

### Questionnaires

Prior to the first session of the experiment, the participants completed a pre-experimental questionnaire that elicited information about their demographics (e.g., age and gender) and exposure to and proficiency in signed and spoken L2s. Following the first session of the experiment, the participants completed a post-experimental questionnaire that asked them to report their previous knowledge of any signs learned in the experiment and whether they accurately followed the instructions.

### Stimuli

Twenty ASL sign videos representing common nouns were selected from the ASL-LEX 2.0 database (Sehyr et al., 2021; see Appendix A). The iconicity ratings from ASL-LEX were provided by 21 to 37 hearing English L1 non-signers and on the basis of a Likert scale ranging from one (not iconic) to seven (highly iconic) (for details see Caselli et al., 2017). To ensure an even distribution of iconicity ratings across the 20 signs, the signs were chosen within three-tenth increments.

Twenty English words representing common nouns were selected for inclusion as catchwords during the learning phase of the first session of the experiment (see Appendix B). Catchwords were selected based on their comparable frequency and weak semantic relationship to the English translations of the signs learned in the experiment.

### Procedure

This experiment was conducted using the Zoom videoconferencing application. Stimuli were presented via screen sharing using PsychoPy (Peirce, 2022; Peirce et al., 2019). Two experimental sessions were administered one week apart. In the first session, participants first learned the signs, and recall was assessed after five minutes. The second session assessed the recall of signs learned in the first session after one week. The experimenter monitored participants throughout both sessions to ensure that instructions were followed.

**Figure 1.**
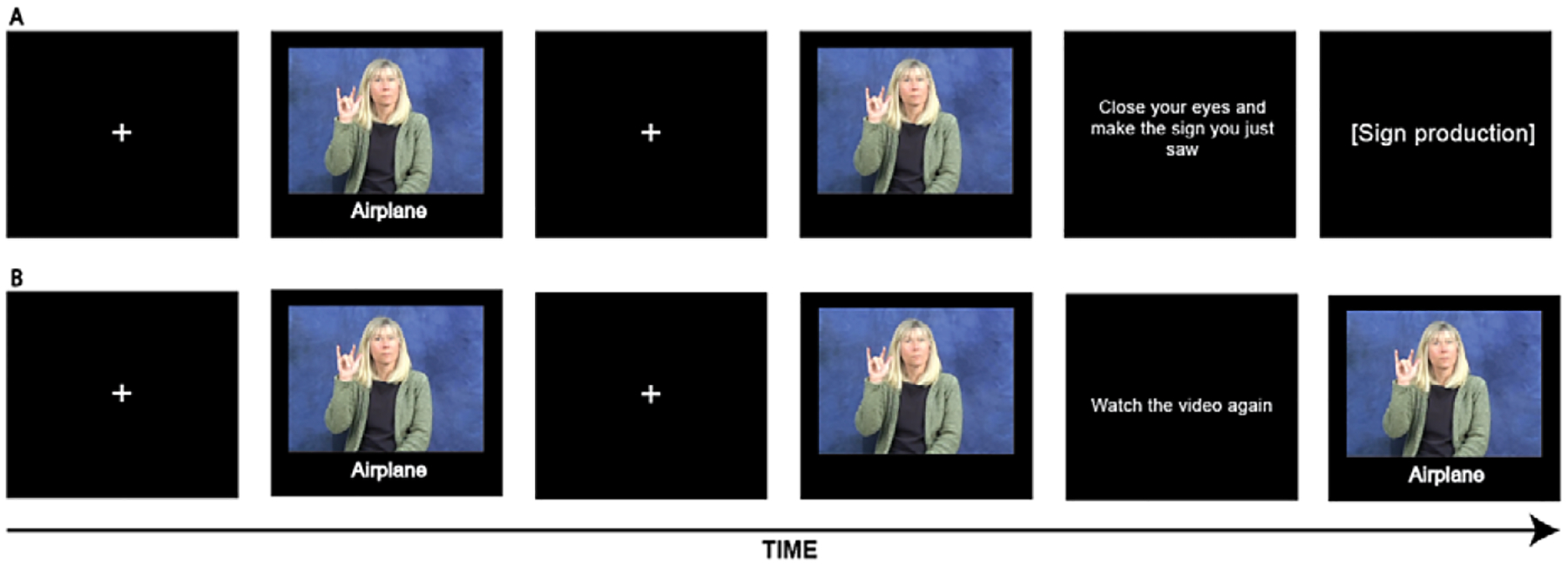
Sample learning trials from (A) production and (B) observation conditions.

The first session consisted of a learning phase and a testing phase. The learning phase consisted of three blocks, during which all the signs to be learned were presented in random order. At the beginning of each learning block, the participants were informed that twenty ASL signs would be presented and that they should try to learn them as best they could. In each trial, a fixation cross appeared for one second, followed by a video of the sign with its English translation underneath. This was followed by a five-second delay to assist in the learning process (Kapnoula & Samuel, 2022). Participants were then instructed either to close their eyes and produce the sign they just saw (production condition) or to be presented with the sign video again without its English translation (observation condition; see Figure 1). This process was repeated for all twenty signs in the set, with the order of presentation randomized in each block for each participant. To eliminate the possible transfer of production to the observation condition, the learning condition was varied between participants such that they learned all signs in either the production condition (*n* = 19) or the observation condition (*n* = 22). To ensure that participants were attending to the experimental task, they were asked to indicate whether they had seen a specific English word in catch trials occurring at four random intervals in each block during the learning phase. This word was either the translation of a sign they had seen or a catchword that they had not seen. After the learning phase, the participants were given a five-minute break before proceeding to the testing phase.

**Figure 2.**
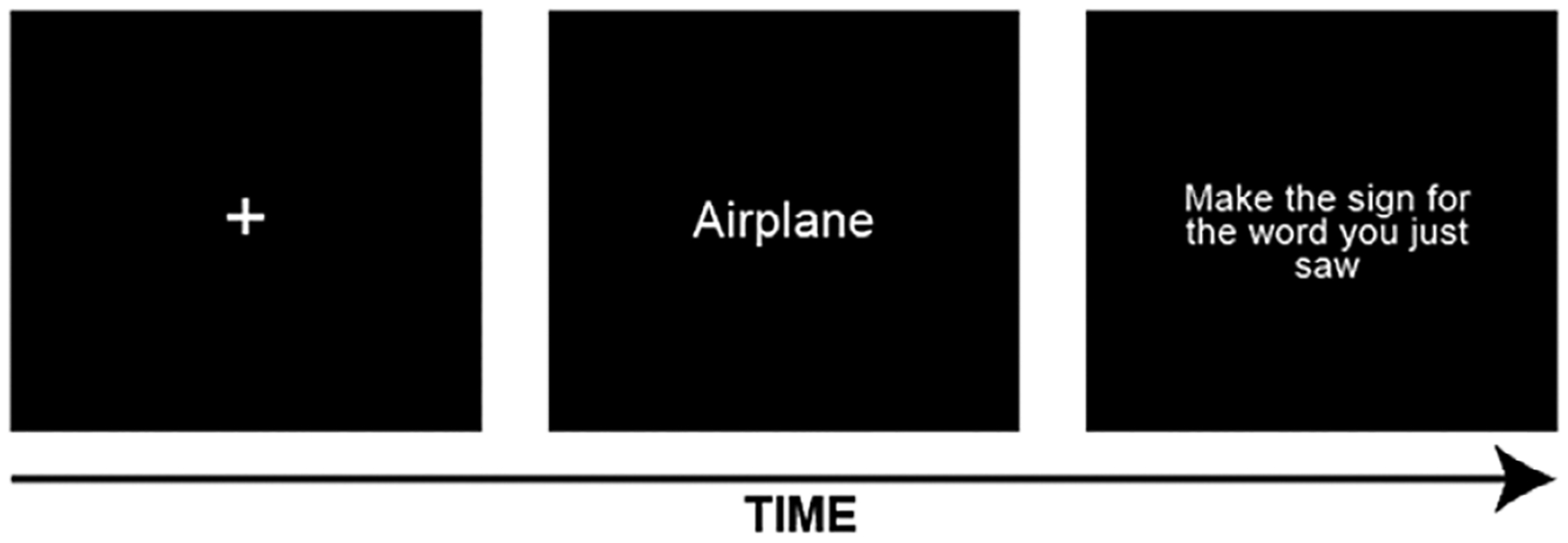
Sample sign recall trial.

The testing phase comprised a sign recall task that tested memory for learned signs via forward translation (English to ASL; see Figure 2). The on-screen instructions stated that English translations of the signs would be presented as text and that participants should produce the corresponding signs as accurately as possible. If a participant did not remember a sign, they were instructed to say so. Knowledge of all twenty ASL signs presented in the preceding learning phase was assessed, and the order of presentation was randomized for each participant. The first session of the experiment lasted approximately thirty minutes.

The second session (one week later) consisted only of the testing phase. All twenty ASL signs learned in the learning phase of the first session were again tested via the sign recall task. The order of presentation was randomized for each participant. The second session of the experiment lasted approximately fifteen minutes.

### Sign recall coding

Sign recall accuracy in the testing phase was coded using three categories: correct (2), partially correct (1), and incorrect (0). ASL signs for which all three phonological parameters (handshape, movement, and location) were produced correctly were coded as correct. Signs for which two of the three phonological parameters were produced correctly (e.g., handshape and location, but not movement) were coded as partially correct. Signs that were forgotten or for which one or zero phonological parameters were produced correctly were coded as incorrect. If a sign was self-corrected, the final production was coded.

The primary coder (a hearing non-signer), who was not involved in data collection and was blind to the learning condition, coded all signs produced by the participants. To establish interrater reliability, a second coder (also a hearing non-signer), who was blind to the learning condition, independently coded all signs produced by eleven randomly selected participants (26.83% of the data). The agreement between the primary and secondary coders was 81%, indicating very good agreement.

### Data analysis

Prior to analysis, any signs that participants reported knowing were excluded for those participants (eight observations), as were signs that participants in the production condition reported watching themselves produce without experimenter knowledge (six observations) and that participants in the observation condition reported producing without experimenter knowledge (two observations). Following these exclusions, sign recall in the testing phase was analyzed in R using a linear mixed effect model via the *lme4* package (Bates et al., 2015), and *p*-values were obtained using the *lmerTest* package (Kuznetsova et al., 2017). Iconicity was grand mean-centered, and categorical fixed effects were coded using weighted mean-centered (Helmert) contrast coding, with the level mentioned first for each factor coded as negative and the level mentioned second coded as positive. The model included fixed effects of learning conditions (observation, production), delay (five minutes, one week), iconicity, and random effects of participant and sign. A data-driven approach to model selection was used such that the maximal random effect structure permitting convergence was used to reduce Type 1 error rates (Barr et al., 2013). Models with random slopes and intercepts were tested to determine whether they converged. If multiple models converged, an ANOVA was conducted to determine whether the model with random slopes accounted for significantly more variance than the model with random intercepts only, and if so, the model with random slopes was reported. De-identified data and analysis scripts are publicly available via the following link: https://osf.io/p8bvr/?view_only=04ca94101c044f0ebdbd1d8f2bcb94fb.

## Results

**Figure 3.**
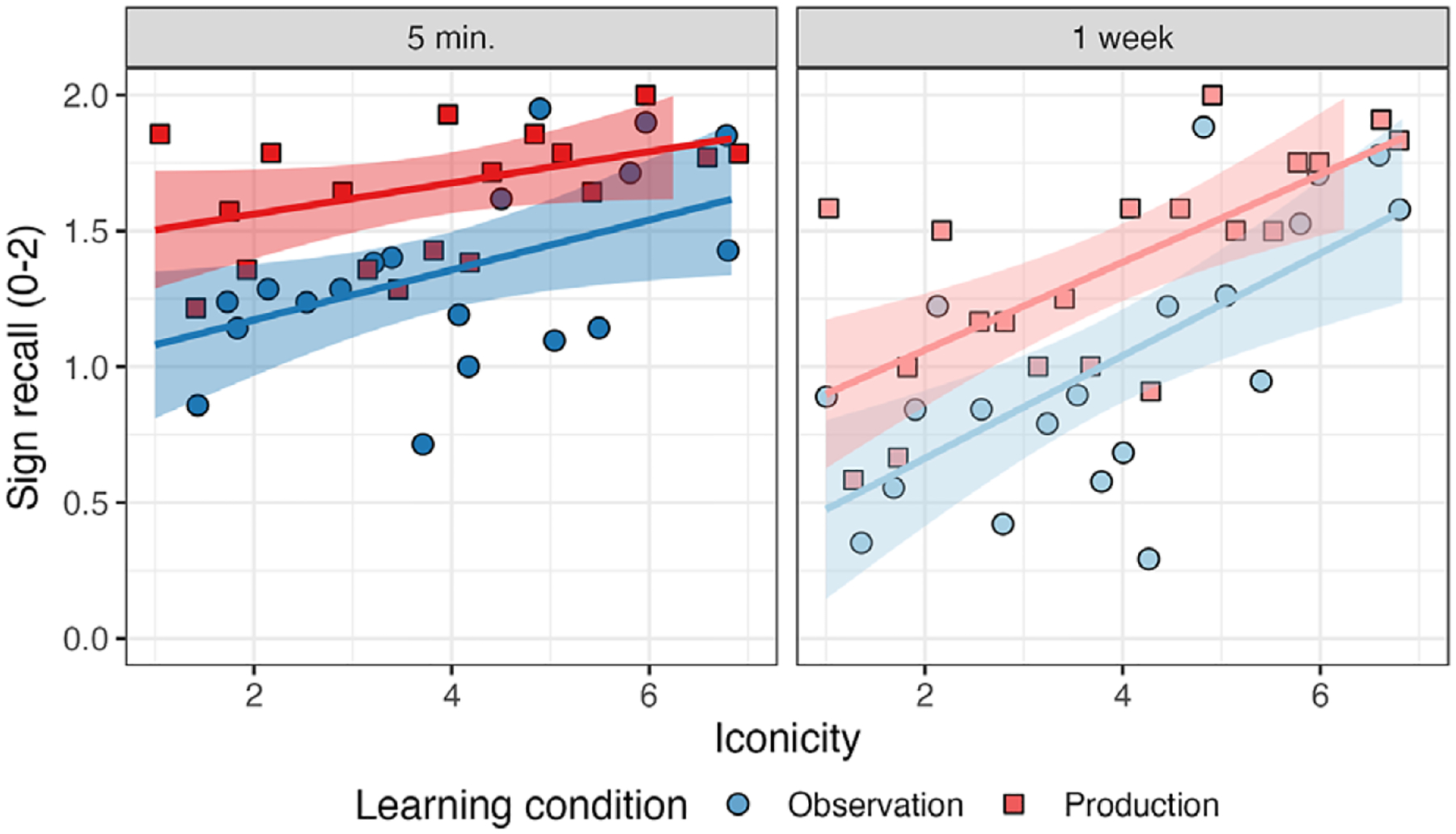
Sign recall by learning condition, delay, and iconicity.

Table 1 displays parameter estimates for the model for sign recall, and Figure 3 displays sign recall by learning condition, delay, and iconicity. Critically, the main effect of the learning condition was significant, indicating that signs learned via production (*M* = 1.55, *SD* = 0.69) were recalled more accurately than signs learned via observation (*M* = 1.21, *SD* = 0.81). In addition, there was a significant main effect of delay, indicating a more accurate recall of signs after five minutes (*M* = 1.47, *SD* = 0.71) than one week (*M* = 1.19, *SD* = 0.83), and a significant main effect of iconicity, indicating that more iconic signs were recalled more accurately than less iconic signs. The interaction between learning condition and iconicity was significant, indicating that as iconicity decreased, the facilitatory effect of production increased. Finally, the interaction between delay and iconicity was significant, indicating that iconicity had a larger effect on sign recall after one week than after five minutes. Consistent with our hypothesis, these results indicate that motor encoding from production facilitates M2L2 sign recall and that this facilitatory effect may be particularly powerful for low iconicity signs.

## Discussion

This study sought to reveal the impact of motor encoding (somatosensory feedback) and the extent to which it interacts with iconicity on M2L2 sign learning. We compared recall accuracy for signs that varied in iconicity when they were learned via motor encoding in an eyes-closed production condition with recall accuracy for signs learned via visual encoding in a sign observation condition. The results show that signs learned in the production condition were recalled more accurately than those learned in the observation condition, providing evidence that motor encoding from production enhances M2L2 sign learning more effectively than visual encoding from observation. Furthermore, the results indicate that signs low in iconicity were recalled more accurately when they were learned in the production condition than in the observation condition, suggesting that motor encoding from production helps compensate for low iconicity. These results are the first to show that motor encoding facilitates M2L2 sign learning in the absence of visual feedback and that motor encoding interacts with iconicity, highlighting the importance of motor encoding as a key mechanism underlying the effect of production on M2L2 sign learning.

Notably, the findings indicate that feeling oneself producing M2L2 signs enhances learning and subsequent recall to a greater extent than seeing someone else produce signs. This result is consistent with previous research showing that producing signs enhances recall of nouns and action phrases similarly to producing actions associated with them (Zimmer & Engelkamp, 2003) and that producing hand gestures conveying the meanings of words from an unfamiliar spoken L2 enhances learning more than observing someone else produce such hand gestures (Garcia-Gamez & Macizo, 2019; Morett, 2018). In both this previous work and the current work, production is executed by oneself, whereas observation involves watching another person’s production. Although we did not include a condition in which participants imagined themselves producing signs or probe whether participants imagined signs prior to production, future work examining the impact of imagining oneself signing may shed light on its impact on M2L2 sign learning. In addition, because of concerns about reliability and demand characteristics, participants were not explicitly instructed to refrain from producing signs between the first and second sessions and were not asked whether they had done so. Therefore, representations of signs may have been strengthened via production between the first and second sessions, further reinforcing the match between the encoding and retrieval contexts, both of which entailed production. Because participants closed their eyes when producing signs, they did not observe themselves signing, suggesting that the beneficial effect of production on M2L2 sign learning may be due to somatosensory feedback rather than visual feedback. This is consistent with previous work showing that deaf signers use somatosensory feedback rather than visual feedback from their own signing to self-monitor and detect errors (Emmorey, Bosworth, et al., 2009). Compared with visual encoding from sign observation, motor encoding may strengthen motoric memory traces for signs, resulting in more accurate sign production during recall. Alternatively, compared with visual encoding, motor encoding during learning may increase the efficiency of sign recall tested via production due to a practice effect, resulting in greater automaticity of sign production. Future research can differentiate between these alternatives by testing memory for signs via recognition in addition to production and by measuring latency in addition to accuracy, providing additional insight into why motor feedback enhances M2L2 sign learning.

As in previous research on M2L2 sign learning (e.g., Campbell et al., 1992), more iconic signs were recalled with greater accuracy than less iconic signs, and the facilitatory effect of iconicity increased over time. This finding complements evidence that iconic gestures facilitate word learning in an unfamiliar spoken L2, particularly over extended delays (Morett, 2014). Thus, the facilitatory effect of iconicity applies to signed and spoken L2 learning. Indeed, more iconic signs may have been more easily guessed than less iconic signs because of the greater resemblance between their physical form and semantic features of their referents, such as iconic gestures. Although sign-gesture resemblance was not examined in the current study, many signs with high iconicity ratings resembled gestures used to convey their referents. Therefore, experience with these gestures may have facilitated sign-referent associations (Ortega et al., 2019) and supported representations of M2L2 signs rather than undermining them, as observed in previous research (Ortega & Morgan, 2015a, 2015b). Whereas previous research demonstrating gesture-sign interference examined immediate sign imitation, the current study examined sign recall in response to English translations following a delay. Furthermore, we coded sign recall accuracy categorically rather than granularly with respect to phonological parameters. In the context of previous research, the positive relationship between iconicity and delayed recall suggests that iconic gestures serve as a firm but imperfect foundation for M2L2 sign representations, which may increase in accuracy with consolidation over time.

The interaction between motor encoding and iconicity suggests that somatosensory feedback from sign production during learning may facilitate M2L2 acquisition of signs low in iconicity by strengthening their motoric memory. In doing so, somatosensory feedback may help compensate for the difficulty in associating low iconicity signs with their English translations, thereby improving recall. Due to the weakness of this interaction relative to other effects observed in the current study and its absence in previous research (Morett, 2015), it will be important to gauge its robustness by determining the extent to which it can be replicated in future research. One way to do so would be to examine memory for M2L2 signs varying in iconicity learned with and without motor encoding via a forced-choice recognition task in which target signs differ minimally from distractor signs in form. This would provide insight into whether the effects of motor encoding, iconicity, and their interaction on M2L2 sign learning observed in the current study generalize to sign recognition based on fine-grained phonological features or are constrained to sign recall based on semantic representations assessed by production.

In conclusion, the results of the current study provide the first evidence that motor encoding from production promotes M2L2 sign recall. Furthermore, it provides the first evidence of an interaction between motor encoding and iconicity, suggesting that motor encoding may compensate for low iconicity in the context of M2L2 sign learning. These findings may be explained by motor encoding, which strengthens motoric memory traces for signs—particularly signs low in iconicity, enhancing their association with referents. These findings have practical implications for M2L2 sign learning, highlighting the benefits of sign production even when signs cannot be observed, particularly for signs low in iconicity. Overall, the results reveal the importance of motor encoding as a key mechanism underlying the effect of production on M2L2 sign learning, demonstrating that feeling signs promote learning and recall.

## Figures and Tables

**Table 1.** Fixed effect (top) and variance estimates (bottom) for the multi-level model for sign recall

Fixed effects	Estimate	CI	p
(Intercept)	1.41	1.26 – 1.56	<0.001***
Learning condition	0.22	0.04 – 0.40	0.017*
Delay	−0.28	−0.37 – −0.20	<0.001***
Iconicity	0.12	0.06 – 0.19	<0.001***
Learning condition × Delay	−0.00	−0.12 – 0.12	1.000
Learning condition × Iconicity	−0.04	−0.07 – −0.00	0.040*
Delay × Iconicity	0.09	0.05 – 0.14	<0.001***
Learning condition × Delay × Iconicity	−0.01	−0.07 – 0.06	0.837
*Random effects*			
σ^2^	0.36		
τ_00 Participant_	0.08		
τ_00 Sign_	0.07		
τ_11 Sign.Delay_	0.02		
ρ_01 Sign_	0.46		
ICC	0.29		
N _Participant_	41		
N _Sign_	20		
Observations	1755		
Marginal R^2^ / Conditional R^2^	0.141 / 0.392		

## Footnotes

1 To address concerns about whether minimal exposure to and knowledge of ASL was a confounding factor, we conducted an alternate analysis excluding all such participants. Despite the sample size falling below the minimum needed for sensitivity to detect interactions, all the main effects and interactions remained significant, except for the interaction between learning condition and iconicity.

## Appendix A ASL Signs Presented in Learning Phase with Non-Signer Iconicity Ratings

**Table:**

Sign	Iconicity rating
Beer	1.33
Excuse	1.67
Culture	1.9
Cherry	2.2
Passport	2.52
Moon	2.83
City	3.15
Audience	3.45
Magnet	3.73
Buffalo	4.00
Envelope	4.21
Mountain	4.5
Lightning	4.87
Hose	5.13
Pole	5.44
Plunger	5.75
Remote	6.00
Piano	6.38
Book	6.68
Two	6.96

## Appendix B English Catch Words Presented in Learning Phase

**Table:**

Lunchroom
Education
Account
Office
Flower
Noise
Doctor
Coast
Skate
Authority
Paper
Cook
Dirt
Sweater
Donkey
Pain
Police
Vessel
Ghost
Funeral
